# Resolution of Inflammation in Neurodegenerative Diseases: The Role of Resolvins

**DOI:** 10.1155/2020/3267172

**Published:** 2020-03-25

**Authors:** Sajad Chamani, Vanessa Bianconi, Aida Tasbandi, Matteo Pirro, George E. Barreto, Tannaz Jamialahmadi, Amirhossein Sahebkar

**Affiliations:** ^1^Birjand University of Medical Sciences, Birjand, Iran; ^2^Unit of Internal Medicine, Department of Medicine, University of Perugia, Perugia, Italy; ^3^School of Medicine, Mashhad University of Medical Sciences, Mashhad, Iran; ^4^Department of Biological Sciences, University of Limerick, Limerick, Ireland; ^5^Health Research Institute, University of Limerick, Limerick, Ireland; ^6^Biotechnology Research Center, Pharmaceutical Technology Institute, Mashhad University of Medical Sciences, Mashhad, Iran; ^7^Department of Nutrition, Mashhad University of Medical Sciences, Mashhad, Iran; ^8^Halal Research Center of IRI, FDA, Tehran, Iran; ^9^Neurogenic Inflammation Research Center, Mashhad University of Medical Sciences, Mashhad, Iran; ^10^School of Pharmacy, Mashhad University of Medical Sciences, Mashhad, Iran

## Abstract

Acute inflammation has been described as a reactive dynamic process, promoted by the secretion of proinflammatory mediators, including lipid molecules like leukotrienes and prostaglandins, and counterbalanced by proresolving mediators including omega-3 polyunsaturated fatty-acid- (PUFA-) derived molecules. The switch from the initiation to the resolution phase of acute inflammatory response is crucial for tissue homeostasis, whereas the failure to resolve early inflammation by specialized proresolving mediators leads to chronic inflammation and tissue damage. Among PUFA-derived proresolving mediators, different eicosapentaenoic acid (EPA) and docosahexaenoic acid (DHA) derivatives have been described, namely, resolvins (resolution phase interaction products), which exert their anti-inflammatory and immune-regulatory activities through specific G-protein-coupled receptors. In recent years, compelling evidence has shown that impairment of resolution of inflammation is a crucial pathogenic hallmark in different neurodegenerative disorders, including Alzheimer's disease and Parkinson's disease. This review summarizes current knowledge on the role of resolvins in resolution of inflammation and highlights available evidence showing the neuroprotective potential of EPA- and DHA-derived resolvins (E-series and D-series resolvins, respectively) in neurodegenerative diseases.

## 1. Introduction

Neurodegenerative diseases, including Alzheimer's disease (AD) and Parkinson's disease (PD), represent a critical threat to human health at a global level. In fact, they are debilitating and largely untreatable conditions whose prevalence is increasing worldwide with aging population. Until two decades ago, the pathogenesis of neurodegenerative diseases was in many respects unclear. In the last years, however, it has been progressively elucidated that they may result from different anomalies in the processing of various neuronal proteins, leading to their abnormal aggregation and accumulation. In addition, compelling evidence has recently shown that inflammation is a crucial pathogenic hallmark in these neurological disorders [1]. However, despite significant progresses that have been made in the knowledge of the pathogenesis of AD and PD, there is still an essential requirement for therapeutic strategies and disease-modifying treatments beyond symptomatic remedies [2].

**Figure 1:**
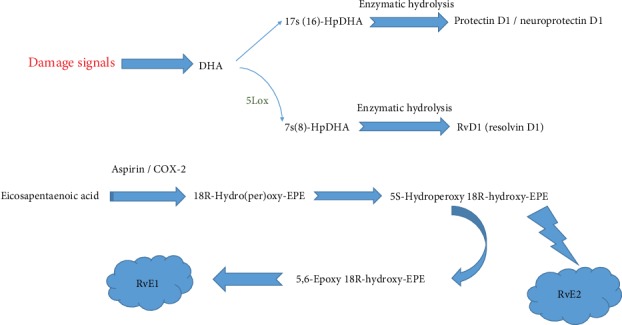
Scheme showing the formation of resolvin. DHA: docosahexaenoic acid; EPA: eicosapentaenoic acid; RvE1: resolvin E1; LOX: lipoxygenases.

Resolvins, different molecules deriving from the lipoxygenase metabolism of eicosapentaenoic acid (EPA), namely, E-series resolvins (RvE), and docosahexaenoic acid (DHA), namely, D-series resolvins (RvD) (Figure 1), are crucial mediators of the resolution phase of acute inflammatory response. In different experimental studies, resolvins have been recognized to inhibit neutrophil infiltration and transmigration [3–7] and to variably modulate the expression of chemokines, adhesion molecules, and other mediators of inflammatory response [8, 9] (Table 1). Therefore, they have attracted attention as possible therapeutic agents in inflammatory conditions, including those affecting the central and peripheral nervous system [10, 11]. Particularly, the potential neuroprotective effects of resolvins attributable to resolution of neuroinflammation have been investigated in neurodegenerative diseases. In this review, we take stock of current knowledge on the role of resolvins in the resolution of inflammation and we highlight available evidence showing the neuroprotective potential of resolvins in AD and PD.

## 2. Resolvins and Resolution of Inflammation

**Figure 2:**
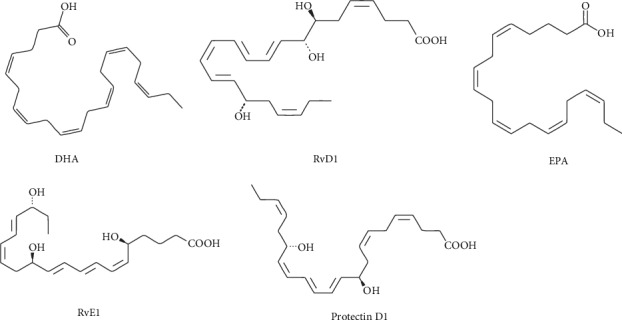
Structure of metabolites. DHA: docosahexaenoic acid; RvD1: resolvin D1; EPA: eicosapentaenoic acid; RvE1: resolvin E1. Citation: https://www.caymanchem.com/product.

In the last years, different oxidized lipid molecules, namely, oxylipins, have been recognized to modulate several biological functions. The term “neuroprotectins” was first used by Serhan and colleagues for a class of oxylipins primarily discovered in neuronal tissues, although the word “protectins” was later implemented when it was found that these compounds were expressed in many other animal tissues. Afterward, oxylipins with equal fundamental characteristics but shaped by different enzymatic reactions were identified and called “maresins.” Subsequently, oxygenated products of two omega-3 polyunsaturated fatty acids (PUFAs), EPA and DHA, were identified and termed “resolvins” or “resolution-phase interaction products,” as they were found to inhibit inflammatory responses (Figures 1 and 2). Due to their close association with inflammation resolution, these lipid metabolites were also referred to as “specialized pro-resolution mediators” (SPMs).

**Figure 3:**
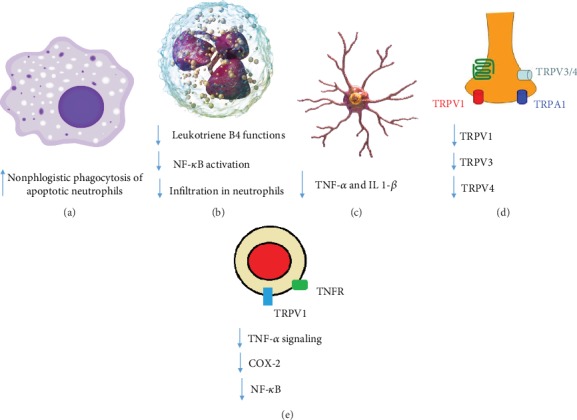
Biological role of SPMs in macrophages (a), neutrophils (b), microglia (c), synapse (d), and monocytes (e).

Although compelling evidence shows that resolvins may exert their powerful anti-inflammatory activities at multiple levels, their main proresolving pathways comprise the modulation of chemotaxis and phagocytic ability of inflammatory cells, along with the control of the expression and activity of a variety of proinflammatory mediators, including arachidonic acid metabolites such as some prostaglandins and leukotrienes [12–15] (Figure 3). Noteworthy, both E-series resolvins (e.g., RvE1 and RvE2), which are bioactive oxygenated lipid products of EPA, and D-series resolvins (e.g., RvD1, RvD2, RvD3, and RvD5), which are DHA derivatives, exert their proresolving action through transmembrane G-protein-coupled receptors (GPCRs). Currently, four receptors for resolvins are known, that is, A lipoxin and formyl peptide receptor 2 (ALX/FPR2), D resolvin receptor 1(DRV1)/GPR32, D-resolvin receptor 2 (DRV2)/GPR18, and chemokine-like receptor 1 (CMKLR1), which is also referred to as ChemR23 or ERV1 [16].

**Figure 4:**
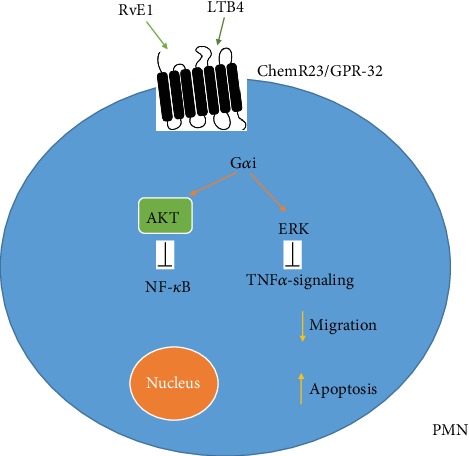
RvE1 blocks NF-*κ*B and TNF*α*-signaling pathways through binding to ChemR23 (chemerin 23) receptor, induces apoptosis, and decreases migration. AKT: protein kinase B; ERK: extracellular signal-regulated kinases; RvE1: resolvin E1.

By binding to ERV1/ChemR23, RvE1 activates a downstream pathway leading to the inhibition of NF-*κ*B signaling in inflammatory cells [17]. Accordingly, the activation of the RvE1-ERV1/ChemR23 axis promotes neutrophil apoptosis and macrophage-mediated phagocytosis, while reducing the production of proinflammatory cytokines (Figure 4) [18]. Available evidence suggests that the proresolving action mediated by RvE2 is more selectively directed towards neutrophils, as compared to that mediated by RvE1. However, whether RvE1 and RvE2 may share the same receptor and signaling cascade remains unclear [16, 19].

D-series resolvins display a variable affinity for three different GPCRs (i.e., ALX/FPR2, DRV1/GPR32, and DRV2/GPR18). RvD1 and RvD3 transduce their signal through both ALX/FPR2 and DRV1/GPR32, whereas RvD2 and RvD5 signal through DRV2/GPR18 and DRV1/GPR32, respectively [16]. The activation of the ALX/FPR2 pathway inhibits the p38 mitogen-activated protein kinase (MAPK) phosphorylation, counteracting the ability of neutrophils and macrophages to migrate and produce proinflammatory mediators [4, 6, 20, 21]. The DRV1/GPR32 signaling not only promotes macrophage-mediated phagocytosis and macrophage polarization toward a proresolution phenotype but also regulates adaptive immune responses by preventing T cell differentiation towards Th1 and Th17 phenotypes and by promoting the generation of regulatory T cells [16]. The RvD2-DRV2/GPR18 axis, beyond being involved in the modulation of neutrophil infiltration ability and on macrophage-mediated phagocytosis, seems to exert a crucial role in the regulation of microglial function [16].

Noteworthy, some resolvin receptors are able to activate different downstream signaling pathways, depending on both the biological context and the presence of additional agonists beyond resolvins. To this regard, it should be emphasized that also some proinflammatory mediators, beyond proresolving mediators, may activate resolvin receptors, leading to the transduction of even opposite biological responses. For instance, chemerin and lipoxin A_4_ may act as proinflammatory ligands of ERV1/ChemR23 and ALX/FPR2, respectively [16]. In addition, some resolvins may bind to other receptors beyond their specific GPCRs, thereby promoting proresolving effects through multiple cellular pathways. For instance, RvE1 may act as a partial agonist of leukotriene B [4] receptor 1 (BLT1), dampening leukotrien-induced proinflammatory signals on leukocytes [22]. Therefore, although resolvins and their receptors have been attracting great attention as possible therapeutic targets against inflammation, a better understanding of their complex pharmacology will be crucial in view of their potential therapeutic use to induce resolution of inflammation in different pathological conditions.

### 2.1. Proresolving Pathways of E-Series and D-Series Resolvins in Different Inflammatory Conditions

The proresolving action of both E-series and D-series resolvins has been reported to exert a crucial preventive/therapeutic role in different inflammatory conditions, including allergic reactions, chronic low-grade inflammation of adipose tissue, ischemia/reperfusion tissue injury, and atherosclerotic plaque formation and progression [16].

In murine models of airway allergic disease, RvE1 has been demonstrated to promote the clearance of eosinophils and antigen-specific T cells, while reducing the expression of proinflammatory cytokines by dendritic cells and Th17 cells [23]. Also, RvD1 has been demonstrated to enhance macrophage phagocytosis and clearance of allergens in a murine model of allergic bronchial reaction [24].

In a mouse model of coronary ligation-induced myocardial infarction, administration of RvE1 has been associated with reduced infiltration of inflammatory cells and reduced production of inflammatory cytokines, with improved recovery of cardiac function [25]. The activation of the DRV2/GRP18 axis has been reported to reduce neutrophil infiltration in a mouse model of hind limb ischemia/reperfusion [26]. In a mouse model of cerebral ischemia/reperfusion injury, exogenous administration of RvD2 reduced infarction area, inflammatory response, and brain edema [27]. In kidney ischemia/reperfusion injury, RvD1 administration reduced infiltrating leukocytes and preserved glomerular function [28].

Individuals carrying a gain-of-function genetic variant of the *ERV1/ChemR23* gene have reduced levels of the inflammatory cytokine IL-6 both in the adipose tissue and in the bloodstream, suggesting that the RvE1-ERV1/ChemR23 axis may be protective against excessive inflammatory burden due to adipose tissue accumulation [29]. Also, RvD1 and RvD2 have been reported to decrease the production of proinflammatory mediators in adipose tissue and to reduce monocyte transadipose migration [30]. Therefore, stimulating the proresolving pathways of both E-series and D-series resolvins may be considered a possible strategy to prevent obesity-related metabolic and cardiovascular complications, which are strictly related to excessive low-grade inflammation.

Lipoxygenase activity, due to its role in the local biosynthesis of resolvins, has been reported to protect mice against atherosclerosis, whereas lipoxygenase deficiency has been shown to promote atherosclerosis progression [31]. In hyperlipidemic mice, the *ERV1/ChemR23* gene deletion has been associated with increased proatherogenic signaling and oxidized low-density lipoprotein uptake by macrophages, as well as reduced phagocytosis and increased necrotic core formation within atherosclerotic plaques [32]. Levels of RvD1 have been demonstrated to be significantly reduced in the vulnerable regions of atherosclerotic plaques of fat-fed low-density lipoprotein receptor (Ldlr)-/- mice [33]. In addition, exogenous administration of either EPA or RvE1 has been associated with reduced atherosclerosis progression in different animal models [32, 34–36]. Similarly, administration of RvD1 to fat-fed Ldlr-/- mice has been shown to promote plaque stability by reducing lesional oxidative stress and necrosis and improving lesional efferocytosis [33]. Therefore, the stimulation of endogenous resolution of inflammation may also represent a potential antiatherosclerotic strategy.

## 3. Neuroinflammation and Neurodegenerative Diseases: The Role of Microglia

The adult human central nervous system (CNS) includes almost 100 billion neurons and an equal amount of glia cells, including astrocytes, oligodendrocytes, and microglia. The CNS parenchyma is separated from the surrounding tissues *via* the blood-brain barrier (BBB), which is made by tight junctions between endothelial cells of the CNS vasculature. The BBB limits and controls the entry of supplements and cells, including peripheral immune cells, in the healthy CNS. This has resulted in the opinion that the CNS is an immune-privileged organ. Nevertheless, this concept has completely changed in recent years, as compelling evidence has shown that the CNS itself is immune-competent and rapidly reacts to damage or infections [2]. In addition, cells of the peripheral innate immune system, including macrophages, can easily pass the BBB under a pathological condition (e.g., BBB breakdown) affecting the CNS (e.g., spinal cord injury, ischemia, and multiple sclerosis). Furthermore, the activation of the peripheral immune system by systemic conditions can accelerate chronic neurodegeneration [37–41].

Although all types of glial cells are of relevance to sustain the homeostasis of the CNS, astrocytes have a crucial role for the trophic support of neurons [42, 43], while oligodendrocytes and microglia act as resident immune cells of the CNS. Under physiological conditions, the so-called resting microglia cells, which are kept resting *via* interacting with neuronal proteins like CX3CL1 (fractalkine) and CD200 [44], monitor the variations of the surrounding CNS environment [45]. However, either systemic or local conditions inducing neuronal damage may lead to microglial cell activation. In case of transient CNS injury, activated microglial cells release neurotrophic factors and promote tissue regeneration [46, 47]. Instead, persistent neuronal injury can lead to the release of proinflammatory cytokines by microglial cells, which in turn may be harmful to the CNS [44, 48]. In fact, tumor necrosis factor- (TNF-) *α* and other inflammatory mediators released by activated microglia can increase the release of reactive oxygen species (ROS), thereby promoting neurodegeneration.

## 4. The Role of Resolvins in Alzheimer's Disease

AD, a neurodegenerative disease that progressively leads to the impairment of cognitive function and skills to execute the simplest jobs, is the leading cause of dementia worldwide. According to the amyloid-*β* (A*β*) theory, the cortical deposition of diverse types of A*β*, due to an imbalance between A*β* production and clearance, is the hallmark of AD neuropathology [49–51]. According to this hypothesis, physiological A*β* elimination, which may occur via transport through the blood-brain barrier (BBB) [52] and enzymatic degradation [53], but also through phagocytosis by microglia [54] and immunomediated mechanisms [55–57], is impaired in AD [58]. However, in the recent years, also, the significant contribution of neuroinflammation to the pathogenesis of AD has been recognized [3, 12–14, 59–67]. To this regard, different studies have shown a significant dysfunction in the resolution of inflammation pathways in AD [68], strongly suggesting proresolving mediators as potential therapeutic strategies. Experimental studies have shown that RvD1 was able to downregulate *β*-amyloid (A*β*) 42-induced inflammation in human microglia [69]. Mizwicki et al. studied the effects of RvD1 on phagocytosis of 6-carboxyfluorescein-labeled A*β* 1–42 (FAM-A*β*) by AD macrophages [70]. In their study, AD macrophage phagocytosis of FAM-A*β* was amplified by RvD1 in a concentration-dependent manner, while caspase-3-positive apoptosis of the AD macrophages stimulated by fA*β* treatment was significantly reduced by RvD1 [70].

A number of experimental studies have shown beneficial effects of PUFA supplementation in terms of reduction of brain A*β* plaque burden or even improvement of cognitive performance in animal models of AD [71–73]. In addition, some clinical studies have investigated the possible therapeutic role of PUFA supplementation in the improvement of cognition in the very early stages of AD [74], showing promising results. Fiala et al. showed that after a 4-17-month PUFA supplementation, the RvD-induced phagocytosis of A*β* by monocytes increased significantly in patients with mild cognitive impairment (MCI) and pre-MCI. However, they did not observe any clinical benefit in terms of cognitive improvement in treated patients [75]. In the OmegAD study (a randomized, double-blind, and placebo-controlled clinical trial), a supplement of 1.7 g DHA and 0.6 g EPA was taken daily for 6 months by AD patients. The analysis of the culture medium of peripheral blood mononuclear cells obtained from treated patients and incubated with amyloid-*β* 1-40 showed unchanged levels of RvD1, which were associated with a stable cognitive status. Instead, a significant decrease of RvD1 levels was seen in the placebo group corresponding to a significant decline in cognitive function [76], suggesting a possible correlation between resolvin expression and cognitive impairment.

## 5. The Role of Resolvins in Parkinson's Disease

PD is a highly prevalent neurodegenerative disease which primarily affects dopaminergic neurons located in the part of the CNS that controls the facilitation of voluntary movements, namely, the substantia nigra [77–79]. The main neuropathological finding in PD is the accumulation of *α*-synuclein-containing Lewy bodies. However, compelling evidence shows that also an imbalance between neuroinflammatory and proresoving processes is involved in the pathogenesis of PD [80–82]. The neuroinflammatory pathway linked with PD starts with the accumulation of posttranslationally modified *α*-synuclein, which may lead to neuronal cell loss and chronic activation of microglia [83]. Such modifications in the microglial phenotype can modify the mesencephalic substantia nigra pars compacta (SNpc) microenvironment by generating a proinflammatory milieu that promotes PD pathogenesis [84–88]. Accordingly, increased plasma levels of proinflammatory mediators (e.g., TNF*α*, IL-1*β*, IL-2, IL-6, COX-1, COX-2, and iNOS) have been shown to exacerbate the dopaminergic neuron damage in PD [89–91]. In addition, both T helper and cytotoxic lymphocytes, promoting a dynamic adaptive immune response inside the substantia nigra, have been recognized to exert a crucial role in the pathogenesis of PD both in experimental and in clinical studies [51, 92, 93].

The effects of stimulating the resolution of inflammation to slow PD progression still remain poorly explored. To the best of our knowledge, the neuroprotective effects of two DHA-derived resolvins, that is, RvD1 and RVD2, have been investigated in experimental models of PD. In a cellular model of PD (i.e., PC12 cells), RvD1 was reported to inhibit 1-methyl-4-phenylpyridinium ion- (Mpp+-) induced expression of proinflammatory mediators [94]. In a rat model of LPS-induced PD, intrathecal injection of RvD2 in SNpc prevented the activation of the NF-*κ*B pathway, thereby inhibiting microglial dysfunction and dopaminergic neuron injury [95]. In an animal study, RvD2 repressed LPS-induced stimulation of glial cells and the onset of defective movements. In fact, LPS-treated rats showed more apomorphine-induced rotational cycles, while rats treated with 25, 50, and 100 ng/kg RvD2 displayed a considerable reduction in the numbers of apomorphine-induced rotational cycles [95]. In the same study, it was shown that RvD2 inhibited LPS-induced microglial stimulation, as revealed by a significant decrease in the expression of proinflammatory mediators and ROS [95].Growing evidence from experimental studies suggest that PUFA administration, by increasing resolvin bioavailability, may represent a potential therapeutic strategy in PD. In a mouse model of PD, a diet enriched with ethyl-EPA increased cortical dopamine levels, attenuated the striatal dopaminergic turnover, and reduced neuronal apoptosis [96]. In a mouse model of *α*-synucleinopathy, DHA intake significantly increased striatal dopamine concentrations [97]. In an animal partial lesion model of PD, the administration of either DHA (50 mg/kg) or its hydroxylated derivate (DHAH) (50 mg/kg) led to positive results on dopaminergic system, neuroinflammation, and oxidative stress and to a significant improvement in amphetamine-induced rotations and cylinder test [98]. Data from clinical studies on the impact of PUFA supplementation on PD onset and progression are awaited.

## 6. Conclusions and Future Perspectives

Growing proof points to that resolvins have strong anti-inflammatory and proresolving properties. As compelling evidence has recently shown that neuroinflammation exerts a crucial role in the pathogenesis of neurodegenerative diseases, resolvins have attracted attention as potential therapeutic strategies in these pathological conditions.

To date, some experimental studies have evaluated the efficacy of resolvins in decreasing neuronal damage in AD and PD, while few clinical studies have investigated the possible therapeutic role of PUFA supplementation in slowing the progression of MCI toward AD. There is an urgent need to further investigate the potential therapeutic role of resolution of inflammation in neurodegenerative diseases in order to provide an effective therapy to these pathological conditions, which are still considered irreversible and incurable.

## Figures and Tables

**Table 1:** Studies reporting resolvin protective actions.

Resolvins	Condition	Effects	References
E-series	Alzheimer's disease	Treatment with RvE1 and LXA4 reversed the inflammatory process and decreased the neuroinflammation associated with A*β* pathology.	[99]
	Allergy	Enhances T cell and eosinophil clearance; abrogates airway hyperresponsiveness. RvE1 promoted the clearance of eosinophils and antigen-specific T cells, while reducing the expression of proinflammatory cytokines by dendritic cells and Th17 cells.	[23, 100, 101]
	Myocardial ischemia/reperfusion injury	RvE1 reduced infiltration of inflammatory cells and reduced production of inflammatory cytokines, leading to improved recovery of cardiac function.	[25]
	Chronic low-grade systemic inflammation	The activation of the RvE1-ERV1/ChemR23 axis reduced the inflammatory burden of adipose tissue.	[29]
	Atherosclerosis	In hyperlipidemic mice, the ERV1/ChemR23 gene deletion led to increased oxidized low-density lipoprotein uptake by macrophages. Exogenous administration of RvE1 reduced atherosclerosis progression in different animal models.	[32, 34–36]

D-series	Alzheimer's disease	Diminished RvD1 production in human Alzheimer's disease.	[102]
	Parkinson's disease	RvD2 prevents the activation of the TLR4/Nf-*κ*B pathway while RvD1 inhibits Mpp+-induced inflammation in PC12 cells (a cell model of Parkinson's disease).	[103]
	Allergy	RvD1 enhanced macrophage phagocytosis and clearance of allergens in a murine model of allergic bronchial reaction.	[24]
	Tissue ischemia/reperfusion injury	Protect from ischemia-reperfusion-induced kidney damage. The activation of the DRV2/GRP18 axis reduces neutrophil infiltration in a mouse model of hind limb ischemia/reperfusion. In a mouse model of cerebral ischemia/reperfusion injury, exogenous administration of RvD2 reduced infarction area, inflammatory response, and brain edema.	[26–28]
	Chronic low-grade systemic inflammation	RvD1 and RvD2 decrease the production of proinflammatory mediators in adipose tissue and reduce monocyte transadipose migration.	[30]
	Atherosclerosis	Levels of RvD1 are reduced in the vulnerable regions of atherosclerotic plaques of fat-fed low-density lipoprotein receptor (Ldlr)-/- mice. Administration of RvD1 to fat-fed Ldlr-/- mice promotes atherosclerotic plaque stability by reducing lesional oxidative stress and necrosis and improving lesional efferocytosis.	[33]
